# Reprogramming the tumor microenvironment by targeting cytidine deaminase in pancreatic ductal adenocarcinoma tumors: implications for the role of P2Y_6_ receptors

**DOI:** 10.1007/s11302-025-10071-0

**Published:** 2025-02-27

**Authors:** Abdel-Aziz S. Shatat

**Affiliations:** Department of Pharmacology and Toxicology, Faculty of Pharmacy, Al-Azhar University, Cairo, Egypt

**Keywords:** P2X6 receptor, Cytidine deaminase, Pancreatic ductal adenocarcinoma, Immunotherapy resistance

## Abstract

Immunotherapies, such as immune checkpoint inhibitors (ICI), anti-cancer vaccines and adoptive T cell transfer, are promising treatments for cancer patients. However, ICI have not shown therapeutic benefit for most mismatch repair-proficient colorectal and pancreatic ductal adenocarcinoma tumors (PDAC), which are aggressive and deadly (Li et al. in Biomedicines 12:2175, 2024). Tumor metabolism can enhance immunological tolerance, but hinder immune cell function. In a recent publication in *Nature Cancer*, Scolaro et al. (Scolaro et al. in Nature Cancer 5:1206–1226, 2024) showed that cytidine deaminase (CDA) upregulation may play a crucial role in shaping the immunosuppressive landscape of human PDAC and other tumors. CDA targeting in pancreatic cancer cell lines led to reduced tumor growth, weight and total regression after treatment aimed at the programmed cell death protein 1 receptor (PD-1) immune checkpoint protein. CDA inhibition, both genetically and pharmacologically, overcame immunotherapy resistance in PDAC models. CDA targeting in PDAC cells altered the tumor microenvironment (TME), enabling T cells to respond to anti-PD-1. In mice with sgNT and sgCda tumors receiving anti-PD-1 treatment, they reduced the number of CD8^+^ T cells. CDA reduction in cancer cells makes tumors more sensitive to immunotherapy, presumably by overcoming immunosuppressive tumor-associated macrophages (TAMs) and forcing them to adopt an immunostimulatory phenotype. The study also found that cancer cells produce a TME rich in UDP (and UTP) by taking advantage of the CDA-mediated pyrimidine salvage pathway. This setting inhibits the recruitment and activation of CD8^+^ T cells by promoting the infiltration and immunosuppressive characteristics of P2Y_6_ receptor–expressing TAMs.

## Commentary

Antibodies against the immune checkpoint protein, PD-1, which inhibit unchecked T cell responses, have been extensively researched for their potential therapeutic use against cancer [1], but immune checkpoint inhibitors (ICI) in general are not very effective against colorectal cancer and PDAC, despite significant response rates and longer survival in certain subsets [2]. Pancreatic ductal adenocarcinoma tumor (PDAC), with a 9% 5-year survival rate, is aggressive and deadly, with a predicted doubling of incidence by 2030, which would make it the second most common cancer-related death. Less than 20% of patients are eligible for resection at the time of diagnosis since most patients present at advanced stages with locoregional spread and/or distant organ metastases as well as most surgery relapses, making treatments ineffective [2, 3]. Therefore, developing treatments that work against unresectable malignancies or prevent recurrence are urgently needed. Tumor metabolism can enhance immunological tolerance, but it can also hinder the function and destiny of immune cells that infiltrate tumors. CDA is an enzyme that hydrolytically deaminates cytidine to uridine and deoxyuridine, preventing DNA integration in cancer cells [4]. CDA contributes to chemoresistance in cancer treatment, but its role in maintaining the extracellular nucleotide pool and immunotherapy resistance remains unexplored.

To find metabolic genes involved in immunotherapy resistance, Scolaro et al. firstly performed a meta-analysis of mouse RNA-sequencing datasets and publicly accessible pretreatment transcriptome datasets of tumors responsive and resistant to ICI, such as anti-CTLA-4 and anti-PD-1. Regarding metabolic genes, the top 10 possibilities were ranked, with CDA being the most important. This study discovered that in comparison to normal tissue, CDA expression was highly elevated in colon, gastric and esophageal malignancies, as well as strongly up-regulated in pancreatic tumors. Moreover, CDA expression was minimal in macrophages and mostly observed in endothelial and cancer cells. This implies that CDA upregulation in cancer cells and its association with ICI resistance may impair the effectiveness of immunotherapy and antitumor responses [5].

According to this study, immunosuppression and CDA expression are correlated in people with PDAC. The PDAC tumors chosen for the investigation had a low immunogenic landscape profile and a high immunosuppressive profile, exhibiting a low expression of a CD8^+^ T cell signature and a significant enrichment of a macrophage signature. CDA expression corresponded with either the CD206^+^ immunosuppressive percentage or tumor border and center CD68^+^ tumor–associated macrophage infiltration in two homogeneous categories of PDAC patients. While low CDA expression was associated with lower total and CD206^+^ TAMs, but higher CD8^+^ T cell infiltration, CDA^high^ PDAC showed considerably decreased CD8^+^ T cell infiltration in the tumor center. Collectively, this suggests that CDA upregulation may play a crucial role in shaping the immunosuppressive landscape of human PDAC and other tumors [6].

Scolaro et al. used two mouse PDAC tumor engraftment models, orthogonal KPC tumors and subcutaneous Panc02 tumors, to examine the relationship between CDA expression in cancer cells and ICI resistance in PDAC. The authors demonstrated that the Panc02 model, which lacks the KRAS activation seen in most human PDAC, was resistant to ICI and has more mutations and antigens than human PDAC. Moreover, in both people and mice, CDA is abundant in the cancer epithelial compartment. Using CRISPR-Cas9, the authors genetically targeted CDA in mouse pancreatic cancer cell lines. Compared to resistant control (sgNT) tumors, CDA targeting in Panc02 cells led to reduced tumor growth and weight, as well as total regression after anti-PD-1 treatment. Most interestingly, following anti-PD-1 therapy, the survival rate of KPC FC1245 tumor–bearing mice was markedly increased by genetic suppression of CDA, confirming the theory that CDA targeting overcomes anti-PD-1 resistance.

Next, the authors infused sgCda KPC FC1245 cells orthotopically with a cDNA encoding CDA. This increased tumor weight and mesenteric lymph node metastases to the levels seen in IgG-treated groups, suggesting that CDA re-expression restored anti-PD-1 resistance. To ascertain if pharmacological blockage of CDA may be utilized therapeutically, Scolaro et al. employed cedazuridine (CDZ), a well-known CDA inhibitor. Mesenteric lymph node metastases, end-stage tumor weight and tumor growth were all reduced by combined CDZ and anti-PD-1 therapy. Therefore, these data confirm that in PDAC models, CDA inhibition, both genetically and pharmacologically, overcomes immunotherapy resistance [7].

Most importantly, CDA targeting in PDAC cancer cells alters the TME, allowing T cells to respond to anti-PD-1. In untreated tissue, CD8^+^ T lymphocytes were absent from the tumor center, demonstrating their incapacity to infiltrate the tumor, but CDA-targeted tumors showed an increase in CD8^+^ T cell infiltration. CD4^+^ T cell infiltration was unaffected in sgCda Panc02 tumors, although that of CD206^+^ macrophages was reduced. Thus, immunosuppression was broken and the T cell response to anti-PD-1 was made possible by CDA inhibition [8]. Furthermore, according to a meta-analysis of several tumor types, anti-PD-1 resistance is mediated by CDA induction in cancer cells. When CDA inhibition was coupled with anti-PD-1 treatment, the anti-PD-1 resistant orthotopic YUMM1.7 melanoma cell line had decreased tumor growth and increased CD8^+^ T cell activation. The authors then investigated if overexpressing CDA in the anti-PD-1-responsive colorectal cancer model, MC38, could induce ICI resistance and found that these cells now displayed elevated aggression and resistance to anti-PD-1 therapy due to poor T cell response induction [9]. Thus, increased CDA activity leads to anti-PD-1 resistance in PDAC, melanoma and colorectal cancer cells.

Next, the authors examined the roles of macrophages and CD8^+^ T lymphocytes in CDA-targeted cells. First, they reduced the number of CD8^+^ T cells in mice with sgNT and sgCda tumors receiving anti-PD-1 treatment and discovered that the CDA-depleted cancer cells had lost their ability to attract macrophages and maintain their immunosuppressive phenotype. Additionally, CDA reduction in cancer cells made tumors more sensitive to immunotherapy, presumably by overcoming immunosuppressive TAMs and forcing them to adopt an immunostimulatory phenotype [10].

CDA catalyzes the synthesis of uridine by deaminating cytidine and by administering a supraphysiological quantity of cytidine (100 µM); this study found that intracellular cytidine levels rose and uridine levels fell in CDA-targeted KPC and Panc02cells compared to untargeted cells. Reduced intracellular levels of uracil nucleotides (UMP, UDP and UTP) in the CDA-targeted cells relative to control reflected this decline in uridine synthesis. DNA and RNA synthesis were unaffected by the variation in uracil nucleotides between sgCda and sgNT KPC FC1245 cells. The CDA substrate, cytidine, was detected in mouse serum, tumor interstitial fluid, absolute fetal bovine serum and the culture media of both dying KPC FC1245 cells and macrophages in vitro.

The release of UTP and UDP by cancer cells has been hypothesized to be a determinant factor in CDA-dependent resistance to anti-PD-1 therapy, as CDA is engaged in a pathway leading to the synthesis and release of uracil nucleotides in cancer cells [11]. Moreover, ectonucleotidases, which are widely expressed in endothelial cells, fibroblasts and immune cells, dephosphorylate UTP to UDP after it has been released into the extracellular milieu. Regarding the involvement of purinergic receptors, the P2Y_6_ receptor, which is activated by UDP, is highly expressed in macrophages. P2Y_6_ receptors can be activated via released UDP or UTP-derived UDP. Also, cells use UDP (and UTP) release to recruit P2Y_6_ receptor–expressing macrophages and maintain their immunosuppressive function. M0- or M2-like bone marrow–derived macrophages (BMDMs) have the highest level of P2Y_6_ receptor expression, whereas M1-like BMDMs have the lowest. Only in P2ry6ΔMy mice did anti-PD-1 reduce the orthotopic KPC FC1245 tumor area in vivo, indicating that P2Y_6_ receptors in macrophages are a crucial regulator of ICI resistance. By activating P2Y_6_ receptors in TAMs, CDA induction in cancer cells encourages immunosuppression. A ‘cool’ immunological milieu and elevated CDA levels in cancer cells were linked in a pan-cancer investigation using 16 scRNA-seq datasets [12].

To conclude, this study revealed how cancer cells produce a TME rich in UDP (and UTP) by taking advantage of the CDA-mediated pyrimidine salvage pathway. This inhibits the recruitment and activation of CD8^+^ T cells by promoting the infiltration and immunosuppressive characteristics of P2Y_6_ receptor–expressing TAMs. Finally, this study presents strong evidence that immunotherapy may be improved by blocking this axis in PDAC and other cancer types.

## Data Availability

No datasets were generated or analysed during the current study.
